# Do active learning techniques promote higher academic performance in an online graduate anatomy course?

**DOI:** 10.1002/ase.70066

**Published:** 2025-06-13

**Authors:** L. J. Bradley, K. E. Meyer, M. S. Kerr, S. D. Maddux, A. J. Heck, R. E. Reeves, E. K. Handler

**Affiliations:** ^1^ Department of Radiology Michigan State University East Lansing Michigan USA; ^2^ Department of Physician Assistant Studies and Executive Director of Division of Academic Innovation University of North Texas Health Science Center Fort Worth Texas USA; ^3^ School of Biomedical Sciences, Associate Dean for Undergraduate Education University of North Texas Health Science Center Fort Worth Texas USA; ^4^ Department of Physiology and Anatomy University of North Texas Health Science Center Fort Worth Texas USA; ^5^ Microbiology, Immunology, & Genetics University of North Texas Health Science Center Fort Worth Texas USA; ^6^ Anatomy and Cell Biology The University of Iowa Iowa City Iowa USA

**Keywords:** active learning, e‐learning, higher education, online learning, student performance

## Abstract

Human anatomy is a foundational course in graduate health professional programs. Given the increased enrollment in anatomy courses, along with the development of new technologies, institutions are increasingly compelled to provide alternative course formats, including online learning. Similarly, higher education is increasingly transitioning from traditional lecture‐based learning to methods that allow students to actively participate in their own learning process (i.e., active learning). Active learning has been shown to have many benefits, including enhanced academic performance. Few studies have investigated the impact of active learning techniques on students' academic performance in online graduate anatomy courses. This study investigates differences in examination performance following participation in four active learning techniques (jigsaw, team‐learning module, concept mapping, and question constructing). Specifically, this study investigated whether academic performance differed between learning objectives assigned to students during an active learning activity compared to non‐assigned learning objectives for each unit. One hundred seventy (170) students completed the online anatomy course. Results showed that academic performance generally did not significantly differ between students who participated in active learning (regardless of technique) and a control group. However, students who participated in active learning were found to perform significantly better on examination questions specifically related to the objective they were required to teach compared to the objectives they learned from their peers. The results of this study suggest that the benefits of active learning on academic performance are largely attributable to preparatory processes required for peer teaching, as recipient peer‐learners appear to gain minimal benefits from active learning.

## INTRODUCTION

Human anatomy is widely recognized as a foundational course in the health professions.^1^, ^2^, ^3^ Currently, the high demand for anatomy education among health profession programs has led to an increase in anatomy course enrollments, as evidenced by the more than 450,000 students enrolling in an anatomy course each year in the United States (U.S.) and Canada as of 2016.^4^, ^5^, ^6^ The high demand for anatomy courses has also compelled universities to provide more accessible learning formats, including online courses.^6^, ^7^ For decades, the prevalence of online learning, where teachers and students are physically separated by geographical distance, has steadily increased in higher education, including anatomy courses.^6^, ^8^, ^9^, ^10^, ^11^, ^12^, ^13^, ^14^, ^15^ According to data from the U.S. Department of Education, of the approximately 3.1 million graduate students in the United States, around 1.1 million have enrolled in at least one online course.^15^ Within the health professions, approximately 47% of graduate students have enrolled in at least one online course, with 27% exclusively enrolled in online courses.^16^ Thus, as the demand for online learning continues to rise, many post‐secondary institutions are challenged to determine how best to teach anatomy in an online learning environment.

### Benefits and criticisms of online learning

Online learning is beneficial to students when compared to in‐person learning due to increased convenience and flexibility^9^, ^17^, ^18^, ^19^, ^20^ and decreased enrollment costs.^21^, ^22^, ^23^, ^24^ Additionally, previous research has shown an increase in student satisfaction and performance.^4^, ^18^, ^25^, ^26^, ^27^, ^28^, ^29^, ^30^, ^31^, ^32^ Research has also identified shortcomings of online learning, including less communication between faculty and students, fewer interactions between peers, and a decrease in motivation and academic performance.^33^, ^34^, ^35^, ^36^, ^37^, ^38^, ^39^, ^40^, ^41^, ^42^, ^43^

### Benefits and criticisms of active learning

Along with the transition to online courses, many graduate‐level health professions programs are transitioning their in‐person curricula from traditional lectures (i.e., solely lecture‐focused presentations) to employ more active learning approaches (e.g., flipped classroom models).^44^, ^45^, ^46^, ^47^ Previous research has shown that the implementation of active learning is associated with higher rates of student satisfaction, motivation, positive learning attitudes, retention, engagement, and academic performance.^41^, ^48^, ^49^, ^50^, ^51^, ^52^, ^53^, ^54^, ^55^, ^56^, ^57^, ^58^, ^59^, ^60^, ^61^, ^62^, ^63^, ^64^, ^65^, ^66^, ^67^, ^68^, ^69^, ^70^, ^71^, ^72^ In addition, active learning has narrowed the achievement gap for historically excluded student groups (i.e., black, Hispanic, Asian, Native American, and Pacific Islander) in science, technology, engineering, and mathematics (STEM) subjects.^73^, ^74^, ^75^, ^76^ This outcome is especially important as previous findings suggest that students who retake anatomy courses due to unsatisfactory performance are commonly members of historically excluded student groups.^77^, ^78^ Despite the overwhelming evidence in support of active learning, educators are still apprehensive about implementing active learning techniques in their courses.^41^, ^79^ The low adoption rates of active learning by educators can be attributed to several reasons, including insufficient time to prepare for active learning, limited resources, a lack of departmental support, and students' resistance to active learning.^67^, ^80^, ^81^, ^82^, ^83^, ^84^, ^85^ As online course enrollments grow, anatomy educators have struggled with how best to create an effective model for online anatomy courses that includes an active learning component that is at least equivalent to the traditional laboratory, team‐based environment.^65^

Given the many benefits of active learning, faculty, administrators, and students have requested higher education to offer more online learning opportunities.^86^ Currently, there is limited research on comparing different active learning techniques in an online anatomy course environment. Given the increase in anatomy course enrollments, possibly due to the increased demand for health professionals, it is imperative for anatomy educators engaging in online education to better understand which active learning methods promote improved academic performance, learning attitudes, and knowledge retention.^87^, ^88^, ^89^

## STUDY PURPOSE

Although many studies have reported differences in academic performance with the implementation of active learning in face‐to‐face courses,^53^, ^60^, ^73^, ^74^, ^78^, ^90^, ^91^, ^92^ few studies have identified which active learning techniques, if any, offer the greatest academic performance benefits for online courses.^50^, ^54^, ^56^, ^93^, ^94^, ^95^, ^96^ Previous studies comparing academic performance outcomes across different active learning techniques have found that students performed better with: (i) e‐clicker questions than discussions,^94^ (ii) case‐based learning than team‐based learning,^97^ (iii) team‐based learning than case‐based learning,^95^ (iv) jigsaw approaches than a think‐pair‐share exercise,^96^ and (v) 1‐minute paper activities than group discussions.^54^ Fewer studies have explored similar comparisons in online courses. In those studies, investigators observed improvements in academic performance when different active learning methods were implemented in online courses compared to traditional lecture methods.^65^, ^70^, ^71^ Diaz et al.^65^ and Vedi & Dulloo^69^ published the only investigation on the influence of active learning implementation on student performance in an online anatomy course and found a similar statistically significant improvement in scores. Due to the paucity of information in the literature, more research is needed to explore the effectiveness of active learning in online anatomy courses. This study aims to assess differences in learner academic performance based on four active learning techniques in an online anatomy course to determine whether some active learning strategies are superior to others for distance learning environments.

## MATERIALS AND METHODS

### Participants and course design

This study was conducted at the University of North Texas Health Science Center (UNTHSC) in Fort Worth, Texas. To be included in the study, participants needed to be registered in the Master of Science in Medical Science program during the Spring 2022 semester and enrolled in the asynchronous, online “Structural Anatomy” course. The research study protocol was granted exempt approval by the North Texas Regional Institutional Review Board, Approval Number 1792843.

The “Structural Anatomy” course was separated into five 3‐week units: (1) upper‐limb musculoskeletal, (2) lower‐limb musculoskeletal, (3) head and neck, (4) cardiopulmonary, and (5) gastrointestinal and renal systems. During each course unit, the first 2 weeks were devoted to learning new course material. The third week was designated for the active learning assignment (part 1 was due on Monday, and part 2 was due on Wednesday). Then, following the active learning activities in week 3, students took the closed‐book unit examination (opened Friday and closed Monday). The active learning activities were a part of the course but did not introduce new course material. The active learning activities allowed students to apply the knowledge they gained in weeks one and two of the unit. All active learning activities utilized the discussion board tool in Canvas Learning Management System (LTI integration by Drieam).

At the beginning of the course, students were divided into five groups with 31–36 students per group. Students remained in their assigned group for the entirety of the semester. During each unit, four out of the five groups participated in a different active learning activity in week three, while one group served as a control group (i.e., no active learning techniques for that unit). In each group, students were further divided into subgroups of approximately 9–13 students and assigned a designated objective that pertained to the content of that specific unit. For each new unit, the five groups rotated to a new active learning technique (or served as the control), with subgroups assigned different objectives that reflected the unit's material. Therefore, each student was assigned a group number and a subgroup letter that followed them through each active learning assignment (e.g., a student assigned to Group 1, subgroup A, would be assigned to the 1A activity for each unit) (Table 1). At the end of the course, each group participated in the four active learning activities and served as a control group for one unit. All active learning techniques were worth the same points within the course grading structure.

**TABLE 1:** Group assignments of active learning techniques and assigned objectives per unit.

	Unit 1	Unit 2	Unit 3	Unit 4	Unit 5
**Group 1**	**Jigsaw** **A:** Brachial plexus **B:** Thumb muscles **C:** Upper limb blood flow	**QC** **A:** Lower limb blood flow **B:** Knee ligaments **C:** Lower limb innervation	**CM** **A:** Orbit cranial nerves **B:** Mouth cranial nerves **C:** Larynx cranial nerves	**TLM** **A:** Respiration muscles **B:** Heart blood supply **C:** Heart conduction	**Control**
**Group 2**	**Control**	**Jigsaw** **A:** Lower limb blood flow **B:** Knee ligaments **C:** Lower limb innervation	**QC** **A:** Orbit cranial nerves **B:** Mouth cranial nerves **C:** Larynx cranial nerves	**CM** **A:** Respiration muscles **B:** Heart blood supply **C:** Heart conduction	**TLM** **A:** Foregut neurovascular **B:** Midgut neurovascular **C:** Hindgut neurovascular
**Group 3**	**TLM** **A:** Brachial plexus **B:** Thumb muscles **C:** Upper limb blood flow	**Control**	**Jigsaw** **A:** Orbit cranial nerves **B:** Mouth cranial nerves **C:** Larynx cranial nerves	**QC** **A:** Respiration muscles **B:** Heart blood supply **C:** Heart conduction	**CM** **A:** Foregut neurovascular **B:** Midgut neurovascular **C:** Hindgut neurovascular
**Group 4**	**CM** **A:** Brachial plexus **B:** Thumb muscles **C:** Upper limb blood flow	**TLM** **A:** Lower limb blood flow **B:** Knee ligaments **C:** Lower limb innervation	**Control**	**Jigsaw** **A:** Respiration muscles **B:** Heart blood supply **C:** Heart conduction	**QC** **A:** Foregut neurovascular **B:** Midgut neurovascular **C:** Hindgut neurovascular
**Group 5**	**QC** **A:** Brachial plexus **B:** Thumb muscles **C:** Upper limb blood flow	**CM** **A:** Lower limb blood flow **B:** Knee ligaments **C:** Lower limb innervation	**TLM** **A:** Orbit cranial nerves **B:** Mouth cranial nerves **C:** Larynx cranial nerves	**Control**	**Jigsaw** **A:** Foregut neurovascular **B:** Midgut neurovascular **C:** Hindgut neurovascular

Abbreviations: CM, concept mapping; QC, question constructing; TLM, team‐learning module.

### Active learning techniques

The four active learning techniques implemented in the course were jigsaw, team‐learning module (TLM), concept mapping (CM), and question constructing (QC). Each student participated in all four active learning techniques during the course. All active learning activities contained two phases: a “teaching” and a “learning” portion. The “teaching” phase required students to teach peers about their designated objective for that unit. In contrast, the “learning” phase allowed students to learn from their peers about the peers' different designated objectives. The following section contains information about the active learning techniques and how each was adapted for an online, asynchronous course.

*Jigsaw* is a cooperative learning method that requires students to work interdependently to achieve a common goal.^60^, ^61^, ^63^, ^66^, ^98^ During jigsaw activities, students were asked to learn the material for their designated subgroup objective and develop strategies to teach their peers. Previous research has argued that jigsaw fosters a deeper understanding of the courses' material^60^, ^61^, ^63^, ^66^ and an increase in enjoyment and academic performance of the course content during in‐person courses.^61^, ^63^, ^66^ In the present study, jigsaw was separated into two phases: an “expert” phase and a “teaching” phase. Beginning with the expert phase, students created and recorded a single‐slide, 3‐minute presentation on their assigned subgroup objective. During the teaching phase, students posted their 3‐minute PowerPoint presentation to a shared discussion board for their group in Canvas. Students could then utilize their peers' teaching presentations for the other designated objectives as supplemental material.

*Team‐Learning Module* (TLM) is a collaborative learning strategy modeled after Team‐Based Learning™ (TBL) (Team Based Learning Collaborative, Huntington, WV). During TLM activities, students follow a three‐step process: preparation, an individual readiness assurance test (iRAT), and a team readiness assurance test (tRAT).^55^, ^99^, ^100^ Previous research has found that TBLs (Team Based Learning™) result in an increase in academic performance, understanding of the course materials, and enjoyment during in‐person courses.^53^, ^101^, ^102^, ^103^, ^104^ In the present study, the preparation phase required students to study their designated subgroup objective for that specific course's unit. Following the preparation phase, students' knowledge of their designated objective was assessed by a five‐question iRAT quiz. The iRAT quiz used Canvas Quizzes and required the Respondus Lockdown Browser (Respondus, Inc., Redmond, WA), which locks down the testing environment for improved quiz security. Once the iRAT quiz closed and was locked for viewing, students with the different designated learning objectives then completed a shared 10‐question worksheet (tRAT) that included each of the assigned objectives. This required students to teach their designated objective to their peers so that the shared tRAT worksheet could be answered correctly and submitted to Canvas Assignments by one group member.

*Concept mapping* (CM) is a learning strategy that scaffolds knowledge by connecting novel information to the original topic using diagrams. Furthermore, CM expects students to not only think about the range of concepts related to the main topic but also introduce how those ideas connect.^52^, ^105^, ^106^ CM is associated with a greater understanding of course materials^107^ and an increase in academic performance during in‐person learning.^52^, ^108^ In the present study, CM was separated into two phases. Phase one tasked students to create a PowerPoint concept map connecting course information to their objective. Additionally, students were required to create an open‐ended question based on their concept map for peers with different teaching objectives to answer. Phase two required students to comment on their peers' concept map with the correct answer to the question posed by each map.

*Question constructing* (QC) is an approach that requires students to construct examination‐style questions. QC has been argued to yield greater satisfaction regarding the learning experience during in‐person courses, improvements in higher‐order thinking, and improved academic performance.^56^, ^109^ In the present study, phase one required students to develop and post two original multiple‐choice questions that pertained to their designated objective for that unit. Students were provided with example questions that were related to the levels of Bloom's Taxonomy^110^ and were asked to create two questions from two different Bloom's levels, with the Bloom's Taxonomy level labeled for each question. During phase two, students responded to two of their peers' posted questions.

During each of the five‐course units, one group rotated as the control group and did not participate in any active learning activities for that unit. As the active learning activities were supplemental, the control group had no additional work and only studied the assigned course materials for that unit. As the control group, no points were assigned for an active learning activity.

### Data collection

#### Academic performance

To assess students' academic performance following each active learning activity, scores from each of the five examinations were used. The examinations were administered through Canvas Quizzes and secured through Respondus Lockdown Browser. Each examination consisted of 30 multiple‐choice questions, each worth two points, for a total of 60 points possible. The examination questions were taken from a question bank, which resulted in a different examination for each student. Because each examination was different, this meant there was a varying number of questions on each examination that pertained to the designated objectives that were reinforced during the active learning activities, which is why difficulty indices were calculated for each examination.

#### Survey administration

Six surveys were administered to the students in the online Structural Anatomy course (one pre‐course survey and five interim‐course surveys). Qualtrics software (Qualtrics LLC, Provo, Utah) was used to construct and administer the surveys through the Canvas learning management system (LTI integration by Drieam). No survey incentives were offered since the surveys were required as part of the course.

At the beginning of the semester, students were asked to complete a pre‐course survey comprised of demographic (e.g., gender, race, age) and previous course enrollment questions (e.g., online enrollment, anatomy enrollment, degree attainment). Following the pre‐course survey, students were de‐identified using a randomly assigned six‐digit number. Students were then assigned to their active learning groups. Certain questions on the survey had points associated with the answers to create active learning groups comprised of diverse but comparable backgrounds. These questions included age (25 or younger: 0 points, 26–30: 1 point, 31–35: 1 point, 36 or older: 0 points), completed major degree (biomedical sciences: 2 points, exercise science/kinesiology: 2 points, biology: 1 point, all others: 0 points), completed minor degree (biology: 2 points, all others: 0 points), overall undergraduate grade point average (GPA) (2.0–2.5 GPA: 1 point, 2.6–3.0 GPA: 2 points, 3.1–3.5 GPA: 3 points, 3.6–4.0 GPA: 4 points), additional degrees (master's degree: 1 point), whether the student had previously enrolled in an anatomy class (1 point), whether the student had previously enrolled in an online class (2 points), and whether the student had enrolled in an online anatomy class (2 points). Student pre‐course survey question scores ranged from 1 to 16 points, with higher scores generally reflecting more experience in online and/or science courses and lower scores reflecting less experience. The active learning groups were then assembled by selecting students with a variety of experiences as determined by survey scores. Each active learning group contained 31–36 students, and combined student scores in the group averaged between 80 and 85 points based on the pre‐course survey questions, which allowed for comparable active learning groups.

Following each examination, students participated in an interim‐course survey asking them to identify which active learning technique they used during the most recent unit. After completing the course, students' interim‐course survey responses and the five examination scores were paired together and de‐identified by one author (KM) and sent to a second author (LB) for analysis.

### Data analysis

Statistical analyses were performed using Number Cruncher Statistical System 2016 (NCSS, LLC, Kaysville, UT) with statistical significance defined as *p* < 0.05. Given that the data in this study were not normally distributed, nonparametric Kruskal–Wallis *H*‐tests (i.e., one‐way ANOVA on rank tests) were used to examine differences in academic performance (i.e., five‐unit examination scores) between the four active learning techniques. A grouping variable with two levels was created. Students who scored correctly on the question directly related to the objective they were assigned for the active learning activity were coded as 1. Students who scored that question incorrectly were scored as 0. Nonparametric Kruskal–Wallis one‐way ANOVA on rank tests was also used to examine differences in academic performance based on this new grouping variable. Dunn's post hoc tests were used for pairwise comparisons.^111^ The magnitude of the summary effect size was interpreted using Cohen's recommendations for small (0.2–0.49), medium (0.50–0.79), and large (≥0.80) effects.^112^

## RESULTS

### Student demographics

One hundred seventy (170) graduate students (cis‐female = 108, cis‐male = 60, other = 2; 78 White, 48 Asian, 25 black/African American, 9 Multiracial, 10 “other” non‐White) in the Master of Science in Medical Science program completed each survey (see appendix) and examination.

### Academic performance with active learning techniques

Results of Kruskal–Wallis tests revealed no statistically significant differences (*p* > 0.05) in learners' academic performance scores between any of the five groups (i.e., four active learning; one control) for units 1, 2, 3, and 4 or final course performance. However, during unit 5, a statistically significant difference was identified (*H* = 12.5, *p* = 0.01) with Dunn's post hoc test revealing that QC, jigsaw, and the control groups outperformed the TLM group (*p* = 0.05, effect size = 0.42). Additionally, jigsaw and control groups outperformed the CM group (p = 0.05, effect size = 0.25).

### Difficulty index for each active learning technique

Difficulty indices of multiple‐choice questions are the percentage of learners who answered an item correctly, ranging from 0.0 to 1.0.^113^ As the difficulty index approaches one (1.0), the easier the question is. For units 1, 3, and 4, Kruskal–Wallis tests revealed there were no statistically significant differences regarding difficulty index across all examination questions between active learning techniques (TLM, CM, Jigsaw, control, and QC). However, for unit 2, a Kruskal–Wallis test indicated significant differences (*H* = 9.6, *p* = 0.05, effect size = 0.66). Dunn's post hoc tests indicated that students in TLM had a higher difficulty index than the control, jigsaw, and QC. Additionally, for unit 5, A Kruskal–Wallis test indicated statistically significant differences (*H* = 10.0, *p* = 0.04, effect size = 0.25). Dunn's post hoc tests indicated that the students in the control had a significantly higher difficulty index than CM and QC. Additionally, post hoc tests indicated that the students in jigsaw had a significantly higher difficulty index than QC (*p* = 0.05, effect size = 0.64).

### Academic performance across all examination questions (assigned and non‐assigned objectives)

For units 1, 2, 4, and 5, Kruskal–Wallis tests revealed there were no statistically significant differences regarding academic performance across all examination questions between objective subgroups (i.e., assigned objectives versus not assigned). However, for unit 3, a Kruskal–Wallis test indicated significant differences (*H* = 8.3, *p* = 0.02, effect size = 0.78).

### Difficulty indices based on designated objectives

During all course units, Kruskal–Wallis tests indicated that there were no statistically significant differences between difficulty indices for the designated objectives.

### Student academic performance across all objective‐associated examination questions (assigned and non‐assigned)

Kruskal–Wallis tests indicated statistically significant differences between the objective subgroups for each unit (Table 2). Consistently, across 5 units and 15 objectives, students who taught the content of an objective performed significantly higher on the examination question related to that objective than students who taught the other objectives. These results suggest that the active teaching process leads to stronger knowledge retention and, thus, higher examination performance scores. See Tables 2 and 3 for examples where peer‐teachers frequently outperformed peer‐learners on the content of interest.

**TABLE 2:** Descriptive statistics and Kruskal–Wallis test results of student academic performance across all objectives‐associated questions on exams (assigned and non‐assigned objectives).

	Count of students (*n*)	Mean ± SD
***Unit 1: Upper limb musculoskeletal*** **Objective A: Brachial plexus** Kruskal–Wallis test (*H* = 9.1, ***p*** = 0.01)		
Brachial plexus	48	83.7 ± 17.0
Thumb muscles	43	74.3 ± 17.5
Upper limb blood flow	43	74.8 ± 21.5
**Objective B: Thumb muscles** Kruskal–Wallis test (*H* = 0.6, *p* = 0.8)		
Brachial plexus	33	87.9 ± 33.1
Thumb muscles	30	93.3 ± 25.4
Upper limb blood flow	27	88.9 ± 32.0
**Objective C: Upper limb blood flow** Kruskal–Wallis test (*H* = 3.3, *p* = 0.3)		
Brachial plexus	48	96.2 ± 12.9
Thumb muscles	43	94.0 ± 11.9
Upper limb blood flow	43	97.7 ± 7.3
***Unit 2: Lower limb musculoskeletal*** **Objective A: Lower limb blood flow** Kruskal–Wallis test (*H* = 9.1, ***p* = 0.01**)		
Lower limb blood flow	47	91.5 ± 13.0
Knee ligaments	44	78.7 ± 23.3
Lower limb innervation	45	85.3 ± 18.8
**Objective B: Knee ligaments** Kruskal–Wallis test (*H* = 3.4, *p* = 0.2)		
Lower limb blood flow	46	84.4 ± 27.2
Knee ligaments	43	94.6 ± 14.2
Lower limb innervation	45	89.5 ± 22.3
**Objective C: Lower limb innervation** Kruskal–Wallis test (*H* = 7.6, ***p* = 0.02**)		
Lower limb blood flow	47	81.9 ± 16.8
Knee ligaments	44	84.0 ± 20.9
Lower limb innervation	45	90.0 ± 16.8
***Unit 3: Head and neck*** **Objective A: Orbit cranial nerves** Kruskal–Wallis test (*H* = 15.6, ***p* = 0.04**)		
Orbit cranial nerves	49	93.8 ± 13.9
Mouth cranial nerves	56	90.0 ± 15.3
Larynx cranial nerves	34	81.3 ± 17.9
**Objective B: Mouth cranial nerves** Kruskal–Wallis test (*H* = 8.2, ***p* = 0.02**)		
Orbit cranial nerves	49	96.5 ± 10.1
Mouth cranial nerves	56	97.0 ± 7.1
Larynx cranial nerves	34	90.5 ± 14.9
**Objective C: Larynx CNs** Kruskal–Wallis test (*H* = 1.4, *p* = 0.5)		
Orbit cranial nerves	31	1 ± 0
Mouth cranial nerves	37	97.3 ± 16.4
Larynx cranial nerves	24	1 ± 0
***Unit 4: Cardiopulmonary*** **Objective A: Respiration muscles** Kruskal–Wallis test (*H* = 14.7, ***p* = 0.0006**)		
Respiration Muscles	46	98.4 ± 5.3
Heart Blood Flow	46	87.7 ± 17.6
Conduction System	43	88.7 ± 18.9
**Objective B: Heart blood flow** Kruskal–Wallis test (*H* = 7.7, ***p* = 0.02**)		
Respiration muscles	46	93.1 ± 11.5
Heart blood flow	45	97.5 ± 6.4
Conduction system	43	88.3 ± 19.7
**Objective C: Conduction system** Kruskal–Wallis test (*H* = 5.4, *p* = 0.07)		
Respiration muscles	43	84.9 ± 29.2
Heart Blood flow	43	95.8 ± 12.0
Conduction system	40	95.9 ± 12.9
***Unit 5: Gastrointestinal and renal*** **Objective A: Foregut innervation** Kruskal–Wallis test (*H* = 4.8, *p* = 0.09)		
Foregut innervation	38	1 ± 0
Midgut innervation	40	92.5 ± 21.3
Hindgut innervation	39	93.6 ± 20.5
**Objective B: Midgut innervation** Kruskal–Wallis test (*H* = 7.7, ***p* = 0.02**)		
Foregut innervation	46	81.2 ± 27.5
Midgut innervation	47	95.1 ± 12.3
Hindgut innervation	43	84.1 ± 32.3
**Objective C: Hindgut innervation** Kruskal–Wallis test (*H* = 0.7, *p* = 0.7)		
Foregut innervation	46	93.1 ± 14.6
Midgut innervation	47	88.8 ± 23.6
Hindgut innervation	43	93.4 ± 1.6

*Note*: Score out of 100; *p*‐values significant at alpha = 0.05 in **bold**.

**TABLE 3:** Dunn's test results investigating student academic performance across all objectives‐associated questions on exams (assigned and non‐assigned objectives).

	Brachial plexus	Thumb muscles	Upper limb blood flow
***Unit 1: Upper limb musculoskeletal*** **Objective A: Brachial plexus**			
Brachial plexus	0.0000	2.7616*	2.3789*
Thumb muscles	2.7616*	0.0000	0.3726
Upper limb blood flow	2.3789*	0.3726	0.0000
**Objective B: Thumb muscles** *No significant pair‐wise differences*			
**Objective C: Upper limb blood flow** *No significant pair‐wise differences*			

	Lower limb blood flow	Knee ligaments	Lower limb innervation
***Unit 2: Lower limb musculoskeletal*** **Objective A: Lower limb blood flow**			
Lower limb blood flow	0.0000	2.8531*	1.4994
Knee ligaments	2.8531*	0.0000	1.3480
Lower limb innervation	1.4994	1.3480	0.0000
**Objective B: Knee ligaments** *No significant pair‐wise differences*			
**Objective C: Lower limb innervation**			
Lower limb blood flow	0.0000	1.3795	2.7570*
Knee ligaments	1.3795	0.0000	1.3473
Lower limb innervation	2.7570*	1.3473	0.0000

	Orbit CNs	Mouth CNs	Larynx CNs
***Unit 3: Head and neck*** **Objective A: Orbit CNs**			
Orbit CNs	0.0000	1.3521	3.9134*
Mouth CNs	1.3521	0.0000	2.8011*
Larynx CNs	3.9134*	2.8011*	0.0000
**Objective B: Mouth CNs**			
Orbit CNs	0.0000	0.2529	2.6472*
Mouth CNs	0.2529	0.0000	2.4901*
Larynx CNs	2.6472*	2.4901*	0.0000
**Objective C: Larynx CNs** *No significant pair‐wise differences*			

	Respiration muscles	Heart blood flow	Conduction system
***Unit 4: cardiopulmonary*** **Objective A: Respiration muscles**			
Respiration muscles	0.0000	3.4795*	3.1193*
Heart blood flow	3.4795*	0.0000	0.3011
Conduction system	3.1193*	0.3011	0.0000
**Objective B: Heart blood flow**			
Respiration muscles	0.0000	1.8671	0.8712
Heart blood flow	1.8671	0.0000	2.7022*
Conduction system	0.8712	2.7022*	0.0000
**Objective C: Conduction system** *No significant pair‐wise differences*			

	Foregut innervation	Midgut innervation	Hindgut innervation
***Unit 5: Gastrointestinal and renal*** **Objective A: Foregut innervation** *No significant pair‐wise differences*			
**Objective B: Midgut innervation**			
Foregut innervation	0.0000	2.7778*	1.4309
Midgut innervation	2.7778*	0.0000	1.2918
Hindgut innervation	1.4309	1.2918	0.0000
**Objective C: Hindgut innervation** *No significant pair‐wise differences*			

*Note*: Regular test: Medians significantly different if *z*‐value >1.9600 (in **bold**). Bonferroni test: Medians significantly different if *z*‐value >2.3940 (*).

Abbreviation: CNs, cranial nerves.

## DISCUSSION

This study is one of the first to investigate differences in student performance by different active learning techniques in an online, graduate anatomy course. Results revealed that academic performance was similar regardless of the active learning technique across the active learning and control groups. The findings also indicated that students scored higher on the course content they taught compared to the content they learned more passively as a listening peer.

Although previous research has demonstrated differences in academic performance with the implementation of active learning techniques in face‐to‐face courses,^52^, ^53^, ^54^, ^55^, ^57^, ^58^, ^59^, ^60^, ^61^, ^62^ the current study found that the active learning activities groups and control group performed comparably on the five‐unit examinations. Previous findings comparing students' self‐reported perceptions of learning with their actual learning in an in‐person physics course using active learning activities found that students' perceptions of learning were less favorable in the active learning setting than in the traditional learning environment.^114^ Deslauriers et al.^114^ attributed these findings to the increased cognitive load required for active learning, whereby increased cognitive load can impair student motivation, commitment, and willingness to engage in their own learning, resulting in a lack of academic performance improvements.^64^ Additionally, Lamon et al.^64^ implemented various active learning activities in a postgraduate nutritional biochemistry and physiology course and found no improvements in academic performance. However, students' satisfaction levels increased. Lamon et al.^64^ and the current study suggest that further research is needed to explore the benefits of active learning in an online course and to better understand what key features cause improvements in course performance.

The current study found that students performed significantly better on examination questions related to the specific content they taught (assigned objective), regardless of the implemented active learning technique. This supports previous research indicating that additional practice and/or teaching can result in an increase in student academic performance.^115^, ^116^, ^117^, ^118^, ^119^, ^120^, ^121^, ^122^, ^123^ Fiorella and Mayer^118^ separated their students into a control group (i.e., traditional, passive learning), a preparation group (i.e., students prepared to teach, but did not teach), and a teaching group (i.e., students prepared and taught course information to their peers). Results indicated that the preparation and teaching group performed significantly better than the control group.^118^ Furthermore, results indicated that only the teaching group performed significantly better after 1 week than the control group.^118^ In the current study, although academic performance improved with the course materials that students taught during their active learning activities, there was no improvement in academic performance related to topics taught to them by their peers. These results suggest that the act of creating and teaching the material is a more powerful learning method than revisiting previously learned material, even in an active learning setting. However, previous research has indicated that multiple exposures to course material enhance memory and, therefore, improve academic performance.^124^, ^125^, ^126^ Together, these results suggest that providing active learning activities that allow students to teach course materials is a productive method for improving academic performance and that passively receiving peer instruction does very little to improve academic performance.

These results show that the benefits of active learning techniques on academic performance are primarily attributable to active techniques that require students to teach the content specifically covered by the examination questions. Linton et al.^119^ found that the active learning techniques that improved academic performance were activities that required “learning by explaining,” indicating that students performed best when they had to teach the course material. Other studies support these findings that teaching best improves academic success.^115^, ^116^, ^118^, ^120^ Bruno et al.^120^ investigated peer teaching in a face‐to‐face undergraduate human anatomy course and found that students who frequently practiced peer teaching performed significantly better on examinations. In conjunction with previous literature and this current study, more substantive research should be conducted to better understand the relationship between active learning techniques implemented in an online anatomy course and student academic performance.

## LIMITATIONS AND FUTURE DIRECTIONS

This study had several limitations. First, the question for each designated objective could have differed for each student on the five examinations because the questions were automatically and randomly drawn from a question bank. However, the questions were specifically chosen as they covered the designated objective thoroughly. Although the examinations were not identical, the difficulty index for each student examination and the designated objective was comparable. However, since only difficulty indices were analyzed for the examination questions, rather than all‐report item‐level statistics (e.g., discrimination indices, point‐biserials, or reliability coefficients), the quality of assessment items is unknown for each question and examination. Secondly, technology aversion may have disadvantaged certain students, given the online nature of the active learning techniques and the required use of PowerPoint and Canvas discussion boards. Thirdly, the results from the study may not be generalizable to all courses because anatomy has unique course content. Finally, this study was completed at a single university and with a single cohort of students, limiting its generalizability to other locales and student populations.

The authors of the current study are already investigating the relationship between academic performance and student satisfaction with the active learning activities implemented in this study. They hope to better understand the relationship between satisfaction and academic performance as related to different active learning techniques with the goal of creating the most effective online anatomy instruction. The aim is to identify which active learning techniques are associated with increased enjoyment, knowledge retention, satisfaction, and academic performance as previous research has shown in face‐to‐face courses.^41^, ^48^, ^52^, ^54^, ^57^, ^58^, ^59^, ^61^, ^65^, ^73^, ^75^, ^76^, ^77^ Additionally, the authors of the current study aim to implement the same active learning techniques within an in‐person anatomy course to compare whether students' academic performance and/or satisfaction differs from the results of the current study.

## CONCLUSIONS

As enrollment in online courses, including anatomy, continues to rise, educators must learn to enact effective teaching strategies for online learning environments. In face‐to‐face courses, active learning is one method that has continually shown higher rates of satisfaction, knowledge retention, and academic performance.^41^, ^48^, ^52^, ^53^, ^54^, ^57^, ^61^, ^68^ In this study, results revealed generally no performance differences on examinations between active learning technique groups and the control group. However, students' academic performance on the specific topics they taught during active learning activities was significantly higher. This study provides important evidence regarding the relationship between academic performance and active learning techniques, with higher scores associated with the material students taught during the active learning activities. Further research is needed to better understand the impacts that different active learning techniques have on students' academic performance.

## AUTHOR CONTRIBUTIONS

**L. J. Bradley:** Conceptualization; investigation; funding acquisition; writing – original draft; methodology; validation; visualization; writing – review and editing; software; formal analysis; project administration; data curation; resources. **K. E. Meyer:** Writing – review and editing; methodology; formal analysis; data curation; validation. **M. S. Kerr:** Validation; writing – review and editing; software; formal analysis; data curation. **S. D. Maddux:** Validation; visualization; writing – review and editing; formal analysis; data curation. **A. J. Heck:** Formal analysis; data curation; writing – review and editing. **R. E. Reeves:** Supervision; conceptualization; investigation; methodology; writing – review and editing; formal analysis; project administration. **E. K. Handler:** Supervision; conceptualization; investigation; methodology; writing – review and editing; formal analysis; project administration.

## CONFLICT OF INTEREST STATEMENT

The authors declare they have no competing interests. All authors have read and accepted this manuscript draft.

## Supporting information


Appendix.
