# Supplementary material for: Do active learning techniques promote higher academic performance in an online graduate anatomy course?
Source: Anat Sci Educ. 2025 Jun 13;18(9):972–84. doi: 10.1002/ase.70066 (PMC12413479; doi:10.1002/ase.70066)
Supplement: Supplementary file 1 — Appendix. [file ASE-18-972-s001.docx]

Pre-Course Survey

1. What gender do you identify with?
   1. Cis female
   2. Cis male
   3. Transgender female
   4. Transgender male
   5. Non-binary
   6. Fluid
   7. Other: (fill in the blank)
2. What is your race?
   1. American Indian or Alaska Native
   2. Asian
   3. Black or African American
   4. Native Hawaiian or Other Pacific Islander
   5. White
3. How old are you?
   1. 25 or younger
   2. 26-30
   3. 31-35
   4. 36 or older
4. What was your completed major degree?
   1. Biomedical sciences
   2. Exercise science/kinesiology
   3. Genetics
   4. Biology
   5. Chemistry
   6. Biochemistry
   7. Other: (fill in the blank)
5. What was your completed minor degree, if any?
   1. Psychology
   2. Sociology
   3. Biology
   4. None
   5. Other: (fill in the blank)
6. What was your overall GPA of your undergraduate degree?
   1. 2.0-2.5
   2. 2.6-3.0
   3. 3.1-3.5
   4. 3.6-4.0
   5. Other: (fill in the blank)
7. Do you have an additional degree or special qualification?
   1. Yes
   2. No
8. If yes, what is your additional degree or special qualification?
   1. Master’s degree
   2. Paramedic/EMT
   3. CNA
   4. Pharmacy technician
   5. Scribe
   6. Other: (fill in the blank)
   7. Not applicable
9. Have you ever taken a human anatomy college-level course?
   1. Yes
   2. No
10. Prior to COVID-19, had you ever taken an online course?
    1. Yes
    2. No
11. If you have taken an online course, did you take a human anatomy course online?
    1. Yes
    2. No
    3. Not applicable

Interim Course Survey

1. On a scale of 1-5, how **prepared** did you feel for the exam after completing the active learning technique in this unit?
   1. Very unprepared
   2. Somewhat unprepared
   3. Neutral
   4. Somewhat prepared
   5. Very prepared
2. On a scale of 1-5, how **useful** was this active learning technique for your understanding of the concepts in this unit?
   1. Not at all useful
   2. Minimally useful
   3. Neutral
   4. Somewhat useful
   5. Very useful
3. On a scale of 1-5, considering the time spent on the active learning technique, how **productive** do you feel this active learning technique was in this unit?
   1. Very unproductive
   2. Somewhat unproductive
   3. Neutral
   4. Somewhat productive
   5. Very productive
4. On a scale of 1-5, how **appropriate** do you feel this active learning technique was for the material in this unit?
   1. Very inappropriate
   2. Somewhat inappropriate
   3. Neutral
   4. Somewhat appropriate
   5. Very appropriate
5. On a scale of 1-5, how **likely** are you to use this active learning technique or elements of this active learning technique to study for other courses in the future?
   1. Very unlikely
   2. Somewhat unlikely
   3. Neutral
   4. Somewhat likely
   5. Very likely
6. Is there anything else you’d like to add? Fill in the blank.

Post-Course Survey

1. Rank how well the active learning techniques **prepared** you in each unit. 1=highest; 4=lowest. Options: jigsaw, question constructing, team-learning module, concept mapping.
2. Rank how **useful** the active learning techniques were in understanding the concepts in each unit. 1=highest; 4=lowest. Options: jigsaw, question constructing, team-learning module, concept mapping.
3. Rank how **productive** the active learning techniques were to understand the course material in each unit. 1=highest; 4=lowest. Options: jigsaw, question constructing, team-learning module, concept mapping.
4. Rank how **appropriate** the active learning techniques were for you in each unit. 1=highest; 4=lowest. Options: jigsaw, question constructing, team-learning module, concept mapping.
5. Rank how **likely** you are to use the active learning techniques in future courses. 1=highest; 4=lowest. Options: jigsaw, question constructing, team-learning module, concept mapping.
6. Is there anything else you’d like to add? Fill in the blank.
